# First Insights into the Draft Genome Sequence of the Endophyte *Paenibacillus amylolyticus* Strain GM1FR, Isolated from *Festuca rubra* L.

**DOI:** 10.1128/genomeA.01516-17

**Published:** 2018-01-25

**Authors:** Anja Poehlein, Jacqueline Hollensteiner, Sandra Granzow, Bernd Wemheuer, Stefan Vidal, Franziska Wemheuer

**Affiliations:** a Genomic and Applied Microbiology and Göttingen Genomics Laboratory, Institute of Microbiology and Genetics, University of Göttingen, Göttingen, Germany; b Department of Crop Sciences, Agricultural Entomology, University of Göttingen, Göttingen, Germany

## Abstract

*Paenibacillus amylolyticus* strain GM1FR is an endophyte isolated from aerial plant tissues of *Festuca rubra* L. Here, we report the draft genome sequence (7.3 Mb) of GM1FR containing 6,241 protein-coding genes, some of which are potentially involved in plant growth promotion and biocontrol.

## GENOME ANNOUNCEMENT

Several strains of the genus *Paenibacillus* from the plant endosphere are known as plant growth-promoting bacteria (1, 2). They are able to produce plant growth-regulating substances such as cytokinin (3) and indole-3-acetic acid (4). In addition, some *Paenibacillus* species act as biocontrol agents against various important phytopathogens and pests (1, 2). We sequenced the genome of the endophyte *Paenibacillus* sp. GM1FR to determine its potential as a biocontrol agent.

*Paenibacillus amylolyticus* strain GM1FR was isolated from surface-sterilized aerial tissues of healthy *Festuca rubra* L. plants. Genomic DNA was extracted using the MasterPure complete DNA purification kit (Epicentre, Madison, WI, USA). The obtained DNA was used to generate Illumina shotgun paired-end sequencing libraries. Sequencing was performed employing the MiSeq system with the MiSeq reagent kit version 3 (600 cycles) as recommended by the manufacturer (Illumina, San Diego, CA, USA). Quality filtering using Trimmomatic version 0.32 (5) resulted in 3,268,102 paired-end reads. The *de novo* genome assembly was performed with the SPAdes genome assembler version 3.8.0 (6). The assembly resulted in 67 contigs (>500 bp) and an average coverage of 92-fold. The assembly was validated and the read coverage was determined with QualiMap version 2.1 (7). Gene prediction and annotation were performed using Prokka (rapid prokaryotic genome annotation) version 1.11 (8).

The draft genome of strain GM1FR consisted of 7,281,281 bp with an overall GC content of 45.47%. It harbored 11 rRNAs, 99 tRNAs, and 6,241 protein-coding genes, including 2,454 genes with functional annotation. A phylogenetic analysis based on multilocus sequence typing using four genes (*gapA*, *groEL*, *gyrA*, and *pgi*) (9) revealed that strain GM1FR clusters with the species *P. amylolyticus* (10).

A total of 58 potential gene clusters involved in secondary metabolite production were identified using antiSMASH version 3.0.5 (11). The majority of these clusters showed no or weak similarity to known clusters. Three putative nonribosomal peptide synthetase (NRPS) gene clusters were identified. One cluster with 62% of the genes exhibited similarity to a pelgipeptin biosynthetic gene cluster. Pelgipeptin exhibits antimicrobial activity against many pathogenic fungi and bacteria (12, 13). A lassopeptide gene cluster with 40% of the genes sharing similarity to a paeninodin biosynthetic gene cluster was detected. Paeninodin is pharmacologically relevant, as it provides a wide range of antimicrobial and antiviral activities (14, 15). Finally, a transAT polyketide synthase-NRPS gene cluster was identified with orthologous genes for each of the genes of a paenilarvins biosynthetic gene cluster. Paenilarvins, which are known for having strong antifungal activities, are produced by the honey bee pathogen *P. larvae* (16). However, it has not been determined if strain GM1FR has antifungal activities. The strain *Paenibacillus amylolyticus* GM1FR contains multiple gene clusters assigned to plant growth and protection as well as health promotion.

### Accession number(s).

This whole-genome shotgun project has been deposited at DDBJ/ENA/GenBank under the accession number MKZL00000000. The version described here is the first version, MKZL01000000.
